# COPE-ICD: A randomised clinical trial studying the effects and meaning of a comprehensive rehabilitation programme for ICD recipients -design, intervention and population

**DOI:** 10.1186/1471-2261-11-33

**Published:** 2011-06-17

**Authors:** Selina K Berg, Jesper H Svendsen, Ann-Dorthe Zwisler, Birthe D Pedersen, Pernille Preisler, Lone Siersbæk-Hansen, Mette B Hansen, Rune H Nielsen, Preben U Pedersen

**Affiliations:** 1 Rigshospitalet, The Heart Center, University of Copenhagen, Copenhagen, Denmark; 2 University of Aarhus, Institute of Public Health, Aarhus, Denmark; 3 University of Copenhagen, Institute of Surgery and Medicine, Copenhagen, Denmark; 4 The Danish National Research Foundation Center for Cardiac Arrhythmia (DARC), University of Copenhagen, Copenhagen, Denmark; 5 University of Southern Denmark, Institute of Public Health, Denmark; 6 University of Southern Denmark, Clinical Institute, Denmark

## Abstract

**Background:**

Growing evidence exists that living with an ICD can lead to fear and avoidance behaviour including the avoidance of physical activity. It has been suggested that psychological stress can increase the risk of shock and predict death. Small studies have indicated a beneficial effect arising from exercise training and psychological intervention, therefore a large-scale rehabilitation programme was set up.

**Methods/Design:**

A mixed methods embedded experimental design was chosen to include both quantitative and qualitative measures. A randomised clinical trial is its primary component. 196 patients (power-calculated) were block randomised to either a control group or intervention group at a single centre. The intervention consists of a 1-year psycho-educational component provided by two nurses and a 12-week exercise training component provided by two physiotherapists. Our hypothesis is that the COPE-ICD programme will reduce avoidance behaviour, sexual dysfunction and increase quality of life, increase physical capability, reduce the number of treatment-demanding arrhythmias, reduce mortality and acute re-hospitalisation, reduce sickness leading to absence from work and be cost-effective. A blinded investigator will perform all physical tests and data collection.

**Discussion:**

Most participants are men (79%) with a mean age of 58 (range 20-85). Most ICD implantations are on primary prophylactic indication (66%). 44% is NYHA II. Mean walk capacity (6MWT) is 417 m. Mean perception of General Health (SF-36) is PCS 42.6 and MCS 47.1.

A large-scale ICD rehabilitation trial including psycho-educational intervention and exercise training has been initiated and will report findings starting in 2011.

**Trial Registration:**

ClinicalTrials.gov: NCT00569478

## Background

Treatment with Implanted Cardioverter Defibrillators (ICD) has reduced mortality remarkably during the past 20 years. It has resulted in new, more extensive guidelines for the implantation of ICDs [1]. The average ICD implantation rate in Europe is 140 per million inhabitants (in Denmark 180 per million) and in the USA the rate is considerably higher, 416 per million [2]. Studies have shown that living with an ICD can lead to anxiety, fear of shock and avoidance of situations, places and objects that the patients associate with shock. It often leads to social isolation, avoidance of physical activity, including sexual activity and mood disturbances [3-8]. In addition, it has been suggested that negative emotions could be the cause rather than the result of arrhythmia, and that psychological stress can increase the risk of shock [9-11]. Furthermore, psychosocial status seems to predict mortality [12]. It is unclear if cardiac rehabilitation can reduce the risk of shock or death.

During the past 10 years several, often small, interventional studies have been carried out aiming to improve every day functioning of patients with ICD.

### Exercise training

Patients enrolled in out-patient rehabilitation (OCR) tend to exercise more often, 4 times a week versus - 3 times a week in the case of non-OCR patients, and with a higher intensity 5.3 METS (metabolic equivalent) versus 3.5 METS in non-OCR patients [13]. Exercise training in a rehabilitation format has been shown to be safe [14-17], to improve cardiopulmonary fitness e.g. exercise time [14], METS [18], Peak Vo2 [14-17] and reduce anxiety [14] and improve quality of life [15].

### Psycho-intervention

Psychological [19]/psycho educational [20-22]/cognitive behavioural [11,23,24] interventions have either a group format [24] or an individual format [19,20] with face to face consultations [24] or telephone counselling [11,19,24]. Psychological intervention seems to have a beneficial effect on anxiety and perceived physical health in patients younger than 65 years but may lead to symptom increase in older patients [19]. Psycho-educational intervention seems to reduce anxiety [20,21,25] and enhance knowledge [20]. However in the computer based format there were no beneficial effects [22]. Cognitive behavioural rehabilitation has shown to improve physical health, reduce anxiety [11,23] and depression, and lower unplanned admissions [23]. However in a group format no difference in quality of life was found [24].

### Combined interventions

A few randomized trials have combined exercise training and cognitive behavioural intervention. In a study by Frizelle et al. from 2004, 22 patients were included, half in an intervention group and half in a waiting group, beginning rehabilitation after 3 months. The intervention consisted of a weekly two-hour group meeting over a 6 week period and a follow up telephone call at 9 weeks. Significant improvements in anxiety, depression, quality of life and physical capacity were observed [26]. Similarly Fitchet et al. compared 16 patients randomised either to a 12 week (twice weekly) rehabilitation programme or a waiting group, beginning rehabilitation after 3 months. The intervention consisted of exercise training and psycho-educational intervention in a group format. This resulted in improved exercise time, and reduced anxiety and depression [27].There have been calls for a well-powered study combining psycho-educational intervention and exercise training [21]. We have now developed and completed enrolment for just such a study. The purpose of the COPE-ICD trial, to our knowledge, the largest randomised clinical intervention trial studying ICD rehabilitation, is to describe the effect and meaning of an outpatient programme including psycho-educational consultations and exercise training for patients with ICD. This paper summarises the study design and the baseline characteristics of the participants.

### Hypothesis

Our hypothesis is that the COPE-ICD programme will:

• reduce avoidance behaviour and increase quality of life

• reduce fear of exercise and increase physical capability.

• reduce sexual dysfunction

• reduce the number of treatment-demanding arrhythmias and ICD shocks.

• reduce mortality

• reduce acute re-hospitalization

• reduce sickness absence and improve employment status in the long term

• be cost effective

## Methods/Design

### Trial design

**Figure 1:**
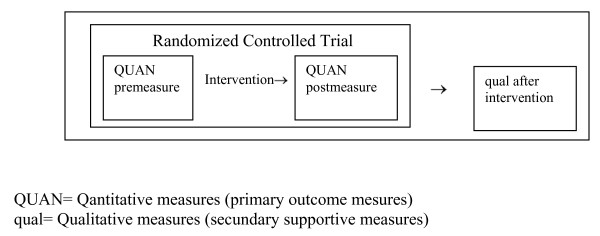
**Trial Design**.

The COPE-ICD trial combines quantitative and qualitative research methods. The methods are integrated by applying a mixed methods embedded experimental design (Figure 1) [28]. The quantitative method utilises the traditional randomised controlled trial (RCT). The qualitative methods utilises extensive interviews of a small selected subset of intervention patients. The qualitative data will help explain the quantitative findings. The rationale for this approach is that the quantitative data and their subsequent analysis provide a general understanding of the research problem. The qualitative data and their analysis refine and explain the statistical results by exploring participants' views in more depth.

### Participants

The setting is a large university hospital with a volume of approximately 300 first time ICD implantations every year.

#### Eligibility

##### Inclusion

- Patients who received an ICD for the first time, and who prior to hospital discharge agreed to participate in the entire programme.

- Patients with physician permission prior to randomization to participate in the physical training programme.

##### Exclusion

- Patients who do not understand the study instructions.

- Patients who are under 18 years of age.

- Patients who are diagnosed with a psychiatric disease.

- Patients with other somatic diseases, where recovery might influence the study.

### Randomisation

Before randomisation baseline data were collected. Patients were randomised while in hospital.

The enrolling research assistant or primary investigator called for randomization allocation. Randomisation (block of 4) was done by voice-respondent in a 1:1 ratio to intervention group or control group (standard care) and there was stratification according to gender and left ventricular ejection fraction (below or above 35%).

### Intervention COPE-ICD

#### Psycho-educational intervention

The programme is based on a humanistic approach, focusing on psychosocial support and education. The programme is directed towards the parameters that ICDs reportedly affect. The content is made up of information and education focused on managing life with an ICD, including emotional reactions (Table 1) using a holistic view on the person and establishment of a joint approach to disease management and coping. Nursing care is inspired by R.R Parse's Human Becoming Practice Methodologies 3 dimensions [29] that are interpreted as: 1, discuss and give meaning to the past, present and future 2, explore and discuss events and possibilities and 3, move along with envisioned possibilities. According to this theory there are 3 ways of changing health: creative imaging that is see, hear and feel what a situation might be like if lived in a different way, affirming personal patterns and value priorities, and shedding light on paradoxes, that is looking at the incongruence in a situation and changing the view held of something. The nurse is true present in the process through discussions, silent immersion and reflection. The consultations take place in a quiet setting at the out-patient clinic. The nurse is able to facilitate contact to or seek advice with a physician or a technician.

**Table 1:** Inspiration Guide for Nursing Consultations

	1	2	3	4	5	6	7	8	9
What happened since you were here last time? How have you been?	x	x	x	x	x	x	x	x	x

Discuss the events leading up to the ICD implant. Experiences before and during admission.	x								

Address present thoughts and questions	x	x	x	x	x	x	x	x	x

Were there any episodes from the ICD?	x	x	x	x	x	x	x	x	x

How did you having an ICD affect your life? Are there any activities you avoid? Are there any places you avoid? Are there objects you avoid?		x							

Did you make an appointment with the physiotherapist/how is training going?			x	x	x	x	x	x	x

Discuss family, how do they tackle, changing patters in the family?				x	x	x			

Information, technical/recommendations.	x				x				

Shock (ad hoc)/phantom shock			x						

Changing view on the body?				x					

Driving		x							

Sexuality, is this affected?				x				x	

The patients consult the nurse in person or by phone once a month for 6 months, and every 2 months thereafter, for the following 6 months. The frequency is based on clinical experience where transportation to the hospital and the need for psycho-educational support are considered. Relatives are invited to participate once or twice if needed. The psycho-educational part of the intervention is performed by two nurses with 10 years of clinical experience each in the care for patients with ICDs. The nurses do not care for ICD patients at the in-hospital unit during the project period to prevent "contamination" of the control group.

#### Exercise training program

Three months after the ICD implantation, training is done twice a week for twelve weeks. The three month delay was chosen to secure stability [20]. The physical training program consists of an individual consultation with the physiotherapist and an individually tailored training programme. A test of aerobic functioning, patients' experiences and usual activities are elements in planning an individual programme. Patients do both resistance and aerobic training, to gain muscle strength and endurance and to gain reduction in after load with a decrease in systemic resistance [30]. The training has either a supervised group format in-hospital or is done at home or in a local hospital rehabilitation setting. The patients use pulse watches while exercising, to ensure that the recommended intensity of the training programme is obtained, and to ensure that the ICD activation threshold is not reached. During the physical training the pulse is noted, at 4 specific times. The patients exercise aerobically, at 50-80% of their estimated maximum pulse, calculated by the Karvonen formula [31]. The target exercise heart rate is close to 80% of their maximum capacity. Patients are encouraged to exercise intensively, to obtain the maximum effect of the training, but not to fatigue themselves. Resistance training is done by 60-80% of one-repetition maximum (1-RM). The training pulse is allowed to rise to a maximum 10 - 15 beats below the ICD activation threshold [30]. To obtain cardiovascular adjustment, it is important to warm up and cool down, to minimize the risk of ischemia and arrhythmia [27,32]. The patients primarily exercise in an upright position, to reduce left ventricular filling pressure and minimize the risk of ischemia or heart failure ventricular arrhythmia [32].

##### Group training at the hospital

The exercise training takes place in a supervised group with planned elements that are individually adjusted with respect to capacity, and risk of injuries.

One session consists of the following; 10 min. warm up, 8 min. biking, 8 min. walking/jogging/running, 8 min. individual aerobic endurance training e.g. step, stairway or running and resistance training of the major muscle groups. The session ends with 10 min. cool down and relaxation. Two physiotherapists with 2-3 years of experience each ran the exercise training programme.

##### Home training

Patients that cannot not attend exercise training at the hospital, are given an individual programme based on the above principles and instructed how to use a pulse watch with extended memory to monitor all training sessions. This is done in order to guide patients and to ensure adherence to instructions.

**Figure 2:**
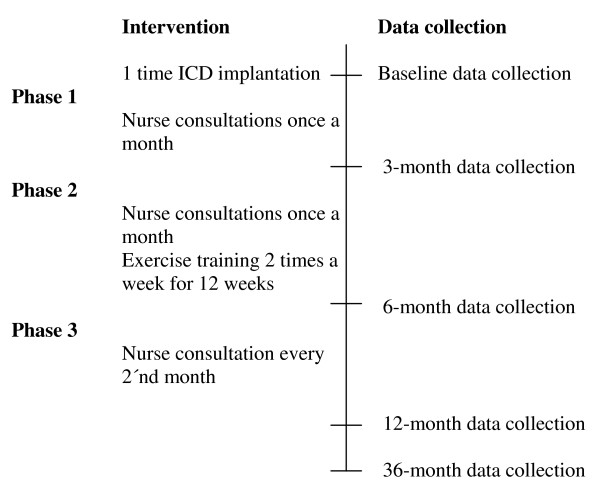
**Timeline**.

An overview of the intervention timeline and data collection is presented in Figure 2.

#### Standard care

Patients in the control group follow the standard care procedure, which is medical follow-up and an invitation to participate in a 2 hour group meeting, which includes information about the ICD and exchange of experiences among patients. In the standard care group no psycho-educational follow-up or exercise training are provided. A bias may occur if patients are offered cardiac rehabilitation at their local hospital. As a national law encourages rehabilitation for all cardiac patients, it is not possible to completely avoid participation in local initiatives. Patients are asked whether they had participated in such activities at the12 months follow-up.

### Outcomes

Using questionnaires, interviews, exercise-tests, a 6-minute walk test (6-MWT), interrogation of the ICD and register-based follow-up, the effect and meaning of the programme are evaluated (Table 2). The pre-training exercise test is performed at 3 months and therefore not reported here.

**Table 2:** Outcome Measures

Measurement	Time
**SF-36**	Baseline, 3-6-12 month

QLI-C	Baseline, 3-6-12 month

IDAS	Baseline, 3-6-12 month

Sex after ICD survey	6 month

ICD and Avoidance Survey	6-12 month

SCA knowledge assessment	Baseline, 12 month

**Exercise test**, 6MWT	3-6 month

ICD interrogation	3-6-12 month

Work	12 month

Rehabilitation elsewhere	12 month

Qualitative interview	12 month

Registry data	2 years

SF-36 and exercise test are primary outcome measures.

Primary outcome measures: Due to the nature of rehabilitation, including both physical and psychological components, the two primary outcome measures are perceived health measured by the Short Form-36 [33,34] questionnaire and Peak VO2, measured by a standardized protocol according to guidelines [35]. The test is performed on bicycle ergometer with an initial workload of 12.5 W and increased 12.5 w every minute until exhaustion or at 5 beats/min heart rate below ICD activation threshold. No gas analyses are conducted.

Secondary outcome measures: Physical capability is measured by an exercise test combined with the six minutes walk tests which have been proved to be effective measures of functional capacity in patients performing cardiac rehabilitation [30,36,37]. Further, exercise training data consist of the number of sessions, duration and heart rate.

Ventricular arrhythmia: 1. ICD-shock 2. Anti-tachycardia pacing is registered by interrogation of the ICD.

Questionnaires: Quality of life is measured by Quality of Life Index-Cardiac version (QLI-C) [38], knowledge of the ICD by Sudden Cardiac Death (SCD) knowledge assessment [20], ICD-adaptation using Implanted Devices Adjustment Scale (IDAS) [39], sexual problems or dysfunction by the Sex After ICD Survey [40] and avoidance behaviour is measured by ICD and Avoidance Survey [40].

Ancillary questions developed for this study: amount of participation in rehabilitation outside the project, and work status/sick leave before and after ICD implantation.

Registry data: Registry data is collected regarding death, hospitalisation, emergency room visits, outpatient visits, medication and employment status. The national registries function very well with a small percentage of lost data and thus are well suited to measure effects, even in small patient populations. Data is extracted from the Danish National Patient Register, the Danish National Health Service Register, the Danish National Prescription Registry and the Danish National Causes of Death Register.

Demographic variables and diagnoses are collected from medical records.

The qualitative study:

The qualitative study seeks to answer the following questions in order to fulfil the purpose of the trial:

Thematic research questions:

- What are the experiences of participating in the COPE-ICD program?

- What is the meaning of participating in the program?

- Which components of the outpatient program are meaningful, and how are they meaningful?

Ten patients (10%) from the intervention group are interviewed at the end of intervention. Qualified interviewees are chosen (knowledgeable, articulate), representative in terms of stratification, gender and left ventricular ejection fraction (LVEF) and according to the reason for ICD implantation (primary/secondary prevention) to achieve maximum variation [41]. The analysis is inspired by Ricoeur's theory of interpretation and consists of three levels: naive reading, structured analysis and critical interpretation and discussion [42].

### Sample size

#### Physical capacity

Based on current knowledge (1), we estimate a difference in V02max of 15% between the rehabilitation group and standard care group (SD = 0.3). A total number of 128 patients are estimated for the trial (alfa = 5%, beta = 20%). *Health status outcome measure: *Based on health status measures among a Danish population compared to patient with heart disease a baseline mean-score of the SF-36 general health (GH) is estimated to be 60 (SD = 22). The effect of rehabilitation is expected to be 10 absolute points. A total number of 154 patients are needed in the trial (alfa = 5%, beta = 20%). The study includes 196 patients, taking a drop-out rate of 20% into account. In the previous trials a drop-out rate around 20% was found, with no significant differences in baseline information on any study variable between groups and no difference in the number of dropouts in each group [11,17,19,21,23].

### Blinding

Because of the nature of rehabilitation, the interventions are open to the staff and the patients. A blinded investigator performs all physical tests, data collection and administration. Blinded outcome analyses are conducted.

### Statistical methods

The statistical analyses of the effects of rehabilitation are conducted based on a predefined statistical protocol using SPSS 17.0 for Windows. The analysis will adopt an analytical approach on all available data, meaning that all data obtained are included in analyses, regardless of adherence to the study protocol. Only data from patients following through the program will be included in the analysis of the endpoints. Accordingly, there is no imputation of missing data. Questionnaire responses will be converted to numerical scores using standard methodologies. Continuous data are checked for normality of distribution, and log-transformed if required. The baseline characteristics will be presented by group to present similarities across treatment, but according to recommendations no significant testing will be performed [43]. The socio-demographic and clinical characteristics of individuals who drop out from the study will be compared with those who completed the study and drop-out characteristics between groups will be compared. The effectiveness of the intervention will be examined using blinded analysis of variance. The primary comparisons will be Two independent samples T-test for the mean difference between two independent samples. Paired T-tests of equality of means will be used for within group analysis of difference over time. Further, univariable and multivariable regression analysis will also be used to investigate the effect of the intervention on the endpoints. The type of regression analysis will depend on the endpoint in question. Demographic, clinical and questionnaire data will be included in multivariable analysis in order to control for their potential confounding effects. The level of significance is set at p < 0.05. Data will, for continues data, be shown as means ± standard deviation or percentage. Categorical data will be shown as percent. Twelve month follow-up data and results obtained from Danish registries regarding hospitalisations are used for follow-up comparison. Due to the skewed distribution of length of hospital stay and repeated measurements of hospital admissions on patients these register-based tertiary outcomes will be analyzed using multivariate random-effect modelling with patients as random effects. Further analysis will be conducted using the Cox regression model including time to first event as outcome variable. At baseline 46% (n = 91) of the population was employed. This is a rather small number of patients and sickness absence will be included as a measure when evaluating employment status. The direct costs of healthcare are calculated by a retrospective top-down method [44]. All other costs are extracted from relevant registers.

### Ethical considerations

The study is approved by the regional Ethics Committee (j.nr. H-B-2007-014), National Agency for Data Security (j.nr. 2007-41-0932) and registered at ClinicalTrials.gov (ID: NCT00569478). Patients were asked to participate in the study after receiving oral and written information, and were given time to reflect on their participation in the study before giving written informed consent. All data are treated in confidence and patients are assured anonymity. The study follows the recommendations of the Declaration of Helsinki II [45].

### Safety

No risks are anticipated during the psycho-educational consultations.

Studies have shown that it was safe for patients with ICDs to participate in physical training [13,16,27]. This intervention is not aimed at maximum performance, but is a recreational programme. A "cool down" period was incorporated in exercise and testing, which has previously resulted in 45,000 exercise tests without complications over a 10 year period [46].

### Participants recruitment and flow

**Figure 3:**
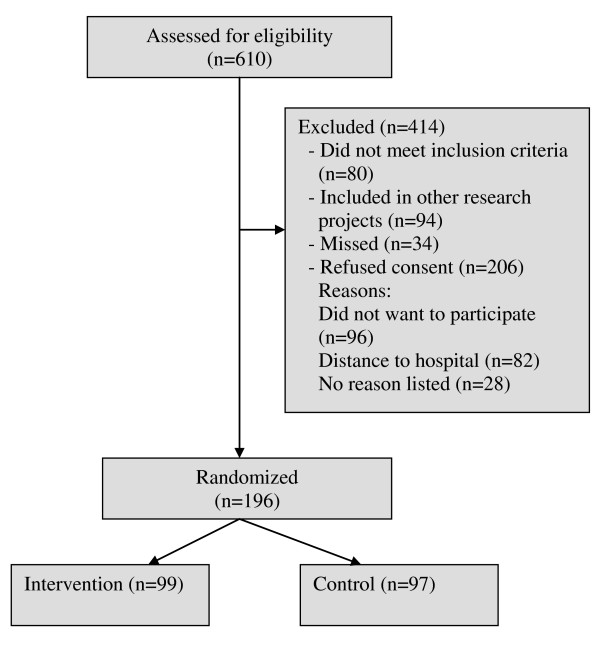
**Flowchart of Patients Included in COPE-ICD**.

During the inclusion period October 2007-November 2009, 610 patients had ICD implantation at the hospital (Figure 3). Due to local policies patients were not asked to participate if they were eligible for or included in pharmaceutical trials. Not meeting the inclusion criteria was due to other somatic diseases (n = 49), psychiatric disease or cognitive dysfunction (n = 20) and language (n = 11). A total of 196 patients were included.

### Baseline measures

Most participants are men (79%) and the mean age was 58 (SD 13.2), range 20-85. The mean LVEF is 32 (SD17.5) and most (46%) are NYHA class 2 and 64% received the ICD for primary prevention (Table 3). The medication status at baseline is presented in Table 4. The mean walk capacity measured by 6MWT is 417 m., Table 5. The perception of health measured by SF-36 is 42.6 for physical component scale (PCS) and 47.1 for mental component scale (MCS), a higher score indicating better perception of health, scale range 0-100 (Table 6). Quality of life score is 26.4 (Table 6), scale range from 0-36, a higher score indicating better quality of life. Baseline knowledge of the ICD score is 20 (Table 6), scale range 0-25.

**Table 3:** Demographic and Physical Profile

	Control gr. n (%)	Intervention gr. n (%)
Male gender	76 (78)	79 (80)

Age (mean)	58	58

Employed	50 (52)	41 (42)

Primary prophylactic indication	67 (69)	63 (64)

VF prior to ICD implantation	20 (20)	21 (21)

LVEF (mean/SD) 73% of tot pop ≤ 35	32.7 (18)	32.2 (17)

NYHA I	18 (19)	30 (31)

NYHA II	44 (46)	42 (43)

NYHA III	32 (33)	24 (25)

NYHA IV	1(1)	2 (2)

BMI ≥ 30	19 (20)	24 (24)

Atial fibrillation	21 (22)	27 (27)

CRT device	9 (9)	14 (14)

History of IHD	57 (59)	45 (46)

Previous MI	33 (34)	20 (21)

Previous PCI	29 (30)	28 (29)

Previous CABG	21 (22)	14 (15)

History of HF	73 (75)	76 (78)

Diabetes	10 (10)	12 (12)

Hypertension	23 (24)	18 (18)

COLD	1 (1)	2 (2)

Other chronic disease	24 (25)	27 (28)

CRT: Coronary resync. therapy. IHD: Ischemic heart disease. MI: Myocardial infarction. PCI: Percutaniuos catheter intervention. CABG: Coronary artery bypass graft. HF: Heart failure. COLD: Chronic Obstructive Lung Disease.

**Table 4:** Baseline Medication

	Control gr. n (%)	Intervention gr. n (%)
Betablockers	83 (86)	76 (76)

Calcium antagonist	6 (6)	18 (18)

Amiodarone	8 (8)	11 (11)

ACE inhibitors	67 (69)	69 (69)

Digoxin	8 (8)	12 (12)

Prolonged nitrates	7 (7)	4 (4)

Insulin	4 (4)	5 (5)

**Table 5:** 6 MWT Baseline

	**Control gr. score**.	**Intervention gr. score**.
Distance meter (SD)	415 (118)	420 (112)

*Shortness of breath Pre-test (CI)	1.1 (0.7-1.3)	0.9 (0.5-1.3)

*Shortness of breath Post-test (CI)	3.2 (2.6-3.8)	2.8 (2.2-3.4)

*Exertion pre-test (CI)	2.1 (1.5-2.7)	1.7 (1.3-2.3)

*Exertion post-test (CI)	2.5 (1.8-3.1)	2.2 (1.5-2.8)

*BORG CR 10 Scale.

Mean values and 95% confidence interval

**Table 6:** SF-36, QoL -cardiac version, SCA knowledge.

	Control gr. score, mean (SD)	Intervention gr. score, mean (SD)
PCS	43.4 (8.5)	41.9 (10.2)

MCS	46.7 (11.5)	47.4 (10.4)

Physical Function	69.8 (22.6)	69.7 (21.4)

Role Physical	39.1 (42.3)	36.6 (42.7)

Bodily Pain	71.2 (27.7)	71.2 (27.1)

General Health Perceptions	59.5 (19.2)	58.7 (21.9)

Vitality	53.5 (22.8)	55.6 (22.9)

Social Functioning	77.7 (24.9)	76.8 (25.9)

Role Emotional	56.3 (39.1)	51.0 (43.7)

Mental Health	69.3 (21.3)	72.5 (20.6)

QLI	26.5 (5.3)	26.3 (6.3)

HFSUBa	23.4 (5.7)	23.5 (6.9)

SOCSUBb	29.9 (6.4)	29.6 (7.1)

PSPSUBc	26.8 (9.4)	26.0 (8.2)

FAMSUBd	30.0 (5.1)	29.6 (8.7)

SCA knowledge	20.1 (3.1)	20 (3.7)

## Discussion

Demographic data and clinical variables are similar between the groups. The baseline characteristics are similar to other ICD rehabilitation populations regarding NYHA, LVEF, gender, type of device and indication [20,21]. The baseline measures of knowledge and skills needed to live with an ICD, SCA Knowledge score 20, are similar to the ICD rehabilitation population that originally tested the questionnaire, score 21 [20]. The population is also similar to other ICD populations regarding quality of life, with very similar scores in all subscales [47,48]. Perceived health measured by SF-36 is also similar to other findings as to the mental and emotional health, but with somewhat higher scores in physical function and pain [48].

Testing 6-MWT showed that this population might have a higher physical capacity (417 m) than other ICD populations (328 m, 383 m) [49,50], which can possibly be explained by a slightly higher LVEF and younger age.

This intervention is expensive from a rehabilitation perspective with 9 nurse consultations, 2 individual consultations with a physiotherapist and 2 weekly exercise training group sessions offered at the hospital. The planned economic analysis will reveal the cost-benefit ratio of this comprehensive rehabilitations programme. The set-up of this programme can be done in any cardiac rehabilitation setting after proper education of nurses and physiotherapists.

Study limitations include the fact that that selection bias may exist due to patients not being included if they were already included in a pharmaceutical trial. The control group might be contaminated by the information given during the project inclusion, suggesting that psycho-educational assistance and exercise training might be beneficial after ICD implantation. This information could lead to control patients seeking rehabilitation elsewhere if they believe it is effective. There is an expected drop-out rate at 20% which could influence the validity. Any difference between patients completing the intervention and those not completing (drop-outs) will be carefully discussed in evaluating the intervention, results and the suitability for implementation. The trial is designed with multiple statistical comparisons so results will be interpreted with caution.

In conclusion, whether a comprehensive rehabilitation programme improves quality of life and physical function have so far not been tested in larger prospective randomized controlled trials. The COPE-ICD trial is designed to test this hypothesis. The baseline data indicate a study population with heart failure, representative for ICD patients in general, similar to previous studies. This is the largest randomized trial in ICD patients to date, and will help determine the value of a comprehensive rehabilitation programme. Furthermore, the study's results will undoubtedly impact on rehabilitation practice and the control group in this study will provide valuable information on the current natural history of contemporary ICD patients. The first results will be published in 2011.

## Abbreviations

ICD: Implantable cardioverter defibrillator; COPE-ICD: Copenhagen Outpatient ProgrammE; METS: metabolic equivalent.

## Competing interests

The authors declare that they have no competing interests.

## Authors' contributions

SKB in collaboration with JHS, ADZ, PUP, BDP designed the study. SKB in collaboration with LSH, PP, MBH, RHN provided the basis for the intervention. SKB drafted the manuscript. All revised the manuscript critically. All have given their final approval of the version to be published.

## Pre-publication history

The pre-publication history for this paper can be accessed here:

http://www.biomedcentral.com/1471-2261/11/33/prepub
